# CDK4/6 inhibitor resistance in estrogen receptor positive breast cancer, a 2023 perspective

**DOI:** 10.3389/fcell.2023.1148792

**Published:** 2023-03-22

**Authors:** Fiona H. Zhou, Teesha Downton, Allegra Freelander, Joshua Hurwitz, C. Elizabeth Caldon, Elgene Lim

**Affiliations:** ^1^ Garvan Institute of Medical Research, Sydney, NSW, Australia; ^2^ St Vincent’s Clinical School, University of NSW, Sydney, NSW, Australia

**Keywords:** estrogen receptor, breast cancer, CDK4/6 inhibitor, endocrine therapy, resistance

## Abstract

CDK4/6 inhibitors have become game-changers in the treatment of estrogen receptor-positive (ER+) breast cancer, and in combination with endocrine therapy are the standard of care first-line treatment for ER+/HER2-negative advanced breast cancer. Although CDK4/6 inhibitors prolong survival for these patients, resistance is inevitable and there is currently no clear standard next-line treatment. There is an urgent unmet need to dissect the mechanisms which drive intrinsic and acquired resistance to CDK4/6 inhibitors and endocrine therapy to guide the subsequent therapeutic decisions. We will review the insights gained from preclinical studies and clinical cohorts into the diverse mechanisms of CDK4/6 inhibitor action and resistance, and highlight potential therapeutic strategies in the context of CDK4/6 inhibitor resistance.

## 1 Introduction

Over 70% of breast cancers are estrogen-receptor positive (ER+) and human epidermal growth factor 2 negative (HER2-) (Howlader et al., 2014). Endocrine therapy (ET) forms the backbone of systemic treatment for this breast cancer subtype. An estimated 20% of patients have innate resistance to ET, and others acquire resistance to ET over a period of time (Anurag et al., 2018). The addition of CDK4/6 inhibitors to ET is efficacious in suppressing cell proliferation, delaying cancer progression and improving survival, and represents the current standard of care first-line therapy for advanced ER+/HER2-breast cancer.

The CDK4/6 inhibitors palbociclib, ribociclib, and abemaciclib are each indicated in the first-line for recurrent unresectable or metastatic ER+/HER2-breast cancer in combination with an aromatase inhibitor (AI), with concurrent ovarian function suppression for pre-menopausal women (Gradishar et al., 2022). There is also data to support the use of combination of a CDK4/6 inhibitor with the selective estrogen receptor degrader (SERD) fulvestrant, particularly in patients with progression on or early relapse after adjuvant AI (Gradishar et al., 2022). Abemaciclib is also FDA approved as monotherapy in refractory advanced disease, having shown single-agent anti-tumor activity in prospective trials (Dickler et al., 2017; Hamilton E. et al., 2022; Hamilton E. P. et al., 2022). More recently, the addition of abemaciclib to adjuvant ET has demonstrated invabsive disease-free survival (IDFS) benefit in high-risk early-stage ER + cancer (Harbeck et al., 2021). The overall survival data is still immature in this abemaciclib trial, as is data from a similarly large phase III study of ribociclib added to AIs in high-risk early-stage ER+ cancer (ClinicalTrials.gov Identifier: NCT03701334).

Palbociclib, ribociclib and abemaciclib differ in biochemical kinase activity assays, their effects on cell proliferation, and on senescence in ER + breast cancer (Fry et al., 2004; Gelbert et al., 2014; Chen et al., 2016; Hafner et al., 2019). All three inhibit the enzymatic activity of CDK4/6 at nanomolar levels and have greater selectivity for CDK4 than CDK6 (Klein et al., 2018; Hafner et al., 2019). Abemaciclib also has the broadest CDK inhibition activity, with additional inhibitory activity against other CDKs including CDK1, 2 and 9 (Hafner et al., 2019). At higher concentrations and with prolonged treatment, abemaciclib has been observed to cause apoptosis (Torres-Guzman et al., 2017), which is in contrast to the cytostatic effect of palbociclib and ribociclib (Jost et al., 2021).

There are also different dosing schedules and toxicity profiles associated with the three agents. They all result in myelosuppression, particularly neutropenia. The incidence of febrile neutropenia however is very low (Braal et al., 2021). Abemaciclib has the least effect on bone marrow suppression and is administered continuously, while patients treated with palbociclib or ribociclib require a 7-day break in the 28-day treatment cycle to allow bone marrow recovery (Chen et al., 2016; Patnaik et al., 2016; Kwapisz, 2017; Klein et al., 2018; Hafner et al., 2019). In contrast, abemaciclib has a greater frequency of gastrointestinal side effects compared to palbociclib and ribociclib (Chen et al., 2016; Kwapisz, 2017; Braal et al., 2021), which may be mediated through CDK9 inhibition (Braal et al., 2021). Prolongation of the QTc interval has been observed with ribociclib, and QTc monitoring after starting treatment is required (Braal et al., 2021).

Other newer CDK4/6 inhibitor agents include dalpiciclib, which has demonstrated a positive progression free survival (PFS) readout in combination with fulvestrant in the second- or third-line setting in the phase III DAWNA-1 trial (Xu et al., 2021), and in combination with an AI in the first-line setting in the phase III DAWNA-2 trial (Xu et al., 2022). Lerociclib had favorable signals of efficacy and tolerability in a dose escalation and expansion trial in combination with fulvestrant (Bulat et al., 2020).

Patients with metastatic disease will eventually progress on the combination therapy due to intrinsic or acquired resistance to the ET, the CDK4/6 inhibitor or both therapies (O'Leary et al., 2018). These scenarios are not differentiated clinically, and currently there is no standard next-line treatment. A better understanding of the mechanisms of CDK4/6 inhibitor and ET resistance and early detection of predictors for resistance may best inform how to sequence or combine currently available and emerging therapies, and potentially identify new drug targets. There is unlikely to be a one size fits all therapeutic approach, and the identification of predictive biomarkers is key to tailoring an individualized next-line treatment plan. Herein we will provide preclinical and clinical perspectives on the management of advanced ER+/HER2-breast cancer following progression on combination ET and CDK4/6 inhibitors.

## 2 Mechanisms of CDK4/6 inhibitor action

### 2.1 CDK4/6 inhibitors induce cell cycle arrest

**FIGURE 1:**
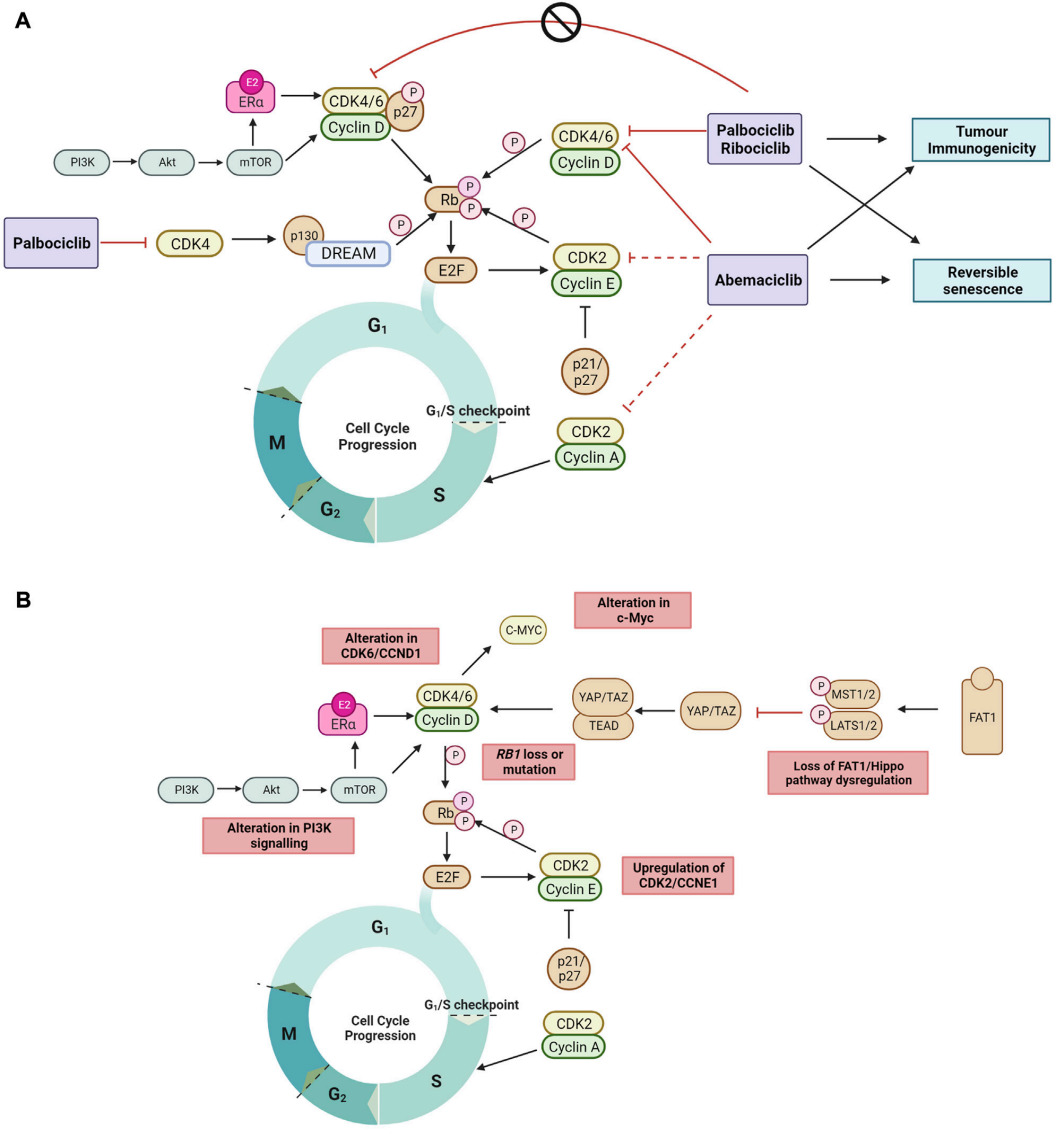
**(A)** The recently proposed mechanisms by which clinical CDK4/6 inhibitors act to block the cell cycle progression to DNA synthesis S phase and G_2_ phase but cannot inhibit tyrosine kinase phosphorylated p27-CDK4/6-CycD complexes in ER + breast cancer (Guiley et al., 2019; Hafner et al., 2019; Schade et al., 2019; Pack et al., 2021). Non-cell cycle effects of CDK4/6 inhibition include reversible senescence (Thangavel et al., 2011; Torres-Guzman et al., 2017; Vijayaraghavan et al., 2017; Marinelli et al., 2020; Maskey et al., 2021; Mayayo-Peralta et al., 2021) and enhanced tumor immunogenicity (Goel et al., 2017; Peuker et al., 2022) **(B)** The mechanisms of ET and CDK4/6 inhibitor resistance currently reported in literature are highlighted. Black arrow shows pathway induction; red line shows pathway inhibition; dotted line indicates lower relative inhibition activity; P, phosphorylation. Images created with BioRender.com.

The best characterized mechanism by which CDK4/6 inhibitors act is the inhibition of retinoblastoma protein (Rb) phosphorylation, leading to G_1_ cell cycle arrest in tumor cells (O'Brien et al., 2018). Palbociclib inhibits growth of both ER+ and ER-negative breast cancer tumors, but only in the context of Rb expression (Fry et al., 2004). Palbociclib inhibition of CDK4 activity blocks the disassembly of the Rb related protein p130 within the DREAM (DP, Rb-like, E2F And MuvB) complex during cell cycle entry (Schade et al., 2019) (Figure 1A), and double Rb and p130 knockout primary fibroblast cells are significantly more resistant to CDK4/6 inhibition compared to Rb knockout cells. This demonstrates that CDK4/6 inhibitors act through Rb and p130, and that a functional Rb axis is required for CDK4/6 inhibitor activity. The loss of functional Rb is a relatively uncommon mechanism of intrinsic resistance (O'Leary et al., 2016; Kumarasamy et al., 2022). While alterations in expression of DREAM complex members are documented in many cancers and correlate with cancer prognosis (Duan et al., 2022; Wang and Liu, 2022), its mechanistic role in CDK4/6 inhibitor resistance is not defined. CDK4/6 inhibition also induces G_1_ cytostatic arrest by generating DNA replication stress (Crozier et al., 2022).

Endogenous CDK inhibitors, including p16, p18, p21 and p27, regulate the cell cycle in healthy cells in response to DNA damage, metabolic changes, and cellular stress (Abukhdeir and Park, 2008; Witkiewicz et al., 2011). However, endogenous CDK inhibitors can hinder the action of synthetic CDK4/6 inhibitors. In ER + breast cancer, overexpression of p16 has been associated with CDK4/6 inhibitor resistance and poor clinical outcome (Palafox et al., 2022). In addition, p18 binds to the CDK6-CycD complex blocking the therapeutic action of CDK4/6 inhibitors (palbociclib or abemaciclib), and conversely CDK4/6 inhibitor sensitivity is restored by suppressing the binding of p18 to CDK6 (Li et al., 2022). The endogenous CDK4/6 inhibitory proteins p21 and p27 prevent phosphorylation by the CDK4-Cyclin D complex, but also facilitate its stabilization and promote its activation. When phosphorylated by tyrosine kinases, p27 allosterically activates the CDK4-Cyclin D complex (Guiley et al., 2019) (Figure 1A). Interestingly, palbociclib has no inhibitory effect on the activity of tyrosine phosphorylated p27-CDK4/6-cycD trimer. Endogenous CDK4/6 inhibitors may also impact the cell cycle indirectly by interfering with CDK4/6 folding which prevents the formation of stable complexes comprising cyclin D, CDK4/6 and p21/p27, thereby releasing p21 to inhibit CDK2 activity and reduce cell proliferation (Guiley et al., 2019; Pack et al., 2021).

### 2.2 Non-cell cycle effects of CDK4/6 inhibitors

When cell proliferation stops in response to contact inhibition, mitogen withdrawal or cytostatic drugs such as CDK4/6 inhibitors, cells exit the cell cycle and enter a quiescent or senescent state. CDK4/6 inhibitors have been shown to induce morphological changes and increase senescence associated (SA)-β-galactosidase activity (Thangavel et al., 2011; Torres-Guzman et al., 2017; Vijayaraghavan et al., 2017; Marinelli et al., 2020; Mayayo-Peralta et al., 2021). This is reversible upon removal of CDK4/6 inhibition, as cells re-enter the cell cycle and resume cell proliferation (Vijayaraghavan et al., 2017), suggesting that CDK4/6 inhibitors do not induce irreversible senescence (Figure 1A). Inhibition of the mTOR signaling complex, mTORC1, during palbociclib exposure has been shown to prevent the induction of permanent senescence, while genetic depletion of TSC2, a negative regulator of mTORC1 during palbociclib exposure resulted in irreversible senescence (Maskey et al., 2021). In addition, abemaciclib and palbociclib treatment have been shown to downregulate mTOR signaling in small cell lung and breast cancer (Naz et al., 2018; Maskey et al., 2021), providing support that CDK4/6 inhibitors may induce reversible senescence through downregulating mTOR signaling.

Enhanced tumor immunogenicity and antitumor immune responses have also been observed with CDK4/6 inhibition (Goel et al., 2017; Deng et al., 2018; Schaer et al., 2018) (Figure 1A). Transcriptomic analysis of serial biopsies from the neoadjuvant NeoPalAna trial (Clinicaltrials.gov identifier NCT01723774) (Ma et al., 2017) showed that palbociclib induced tumor cell expression of endogenous retroviral elements. This results in increased intracellular levels of double-stranded RNA, increased production of type III interferons, and greater tumor antigen presentation. Palbociclib and abemaciclib also suppressed the proliferation of regulatory T cells and promoted cytotoxic T-cell-mediated clearance of tumor cells (Goel et al., 2017). Recently, it was also demonstrated in the RIBECCA trial (Clinicaltrials.gov identifier NCT03096847) that ribociclib treatment resulted in activation of an already existing immune response rather than a *de novo* immune induction in patients with ER + breast cancer (Peuker et al., 2022). Combination therapy of CDK4/6 inhibitors with immune checkpoint blockade has been shown to increase anti-tumor efficacy in preclinical models (Goel et al., 2017; Deng et al., 2018) and provides the rationale to evaluate this combination clinically.

## 3 Pivotal CDK4/6 inhibitor clinical trials in ER + breast cancer

Palbociclib, ribociclib and abemaciclib have demonstrated statistically significant and clinically meaningful PFS benefit when added to ET in both the first and second-line advanced ER+/HER2-breast cancer settings (Cristofanilli et al., 2016; Finn et al., 2016; Sledge et al., 2017; Hortobagyi et al., 2018; Slamon et al., 2018; Johnston et al., 2019). This has translated to overall survival (OS) benefit in pivotal trials with a fulvestrant backbone (Sledge et al., 2020; Slamon et al., 2021; Cristofanilli et al., 2022). In contrast, only ribociclib in combination with an AI have reported an improvement in OS (Hortobagyi et al., 2022; Lu et al., 2022). OS benefit was not seen for first-line palbociclib with letrozole (Finn et al., 2022), and OS results have yet to be reported for first-line abemaciclib with AI (Goetz et al., 2022b).

With the success of CDK4/6 inhibitors in the metastatic setting, they have subsequently been evaluated in the adjuvant setting for early-stage disease. The phase III monarchE trial reported that the addition of 2 years of abemaciclib to adjuvant ET improved invasive disease free (IDFS) survival in high-risk early-stage ER+/HER2-breast cancer (O'Shaughnessy et al., 2021). In contrast, the Penelope-B and PALLAS trials failed to show an IDFS benefit with the addition of 2 years and 1 year of palbociclib respectively to adjuvant ET (Loibl et al., 2021; Mayer et al., 2021). Potential contributing factors to the differing results including differences in study population, duration of CDK4/6 inhibitors, and pharmacological differences between the agents (Loibl et al., 2021; Mayer et al., 2021). The results of the NATALEE trial (Clinicaltrials.gov identifier NCT03701334) assessing the addition of 3 years of ribociclib to adjuvant ET are pending.

## 4 Mechanisms of resistance to combination CDK4/6 inhibitor and endocrine therapy

Other than ER, there are no other routinely used clinical biomarkers used to select patients for combination CDK4/6 inhibitor and ET. ER loss occurs in a minority of ER + breast cancer through the course of therapy, is associated with ET resistance, and remains an important predictor of CDK4/6 inhibitor and ET efficacy (Finn et al., 2020; Griffiths et al., 2021).

Mechanisms of CDK4/6 inhibitor resistance that have been identified are varied and affect cell cycle targets (e.g., Rb1, cyclin E, CDK2, CDK6, c-Myc and AURKA) and/or activated signaling targets (e.g., AKT1, FGFR, HER2, EGFR and RAS) (Wander et al., 2020; Asghar et al., 2022). Only a small percentage of patients are found to harbor each of the individual genomic aberrations that drive resistance at baseline, and a clearer picture of acquired genomic drivers of resistance is emerging with biomarker analysis, in particular circulating tumor DNA (ctDNA) based studies of pivotal CDK4/6 inhibitor trials (Asghar et al., 2022). In some instances, it is also possible that CDK4/6 inhibition delays the onset of endocrine resistance, and that resistance to combination is driven primarily by resistance to the ET backbone (O'Leary et al., 2018). In support of this, ctDNA biomarker analysis of MONARCH-3 trial found a lower incidence of *ESR1* mutations in the abemaciclib plus AI arm compared to the AI alone arm (17% vs. 31% respectively) (Goetz et al., 2020). Here, we list the most well-characterized resistance mechanisms and acquired genomic aberrations identified to date (Figure 1B).

### 4.1 Loss of Rb1

As CDK4/6 inhibitors act to prevent Rb protein inactivation, mutations resulting in a biallelic loss of function in the *RB1* gene have been identified as drivers of resistance to CDK4/6 inhibitors (Herrera-Abreu et al., 2016; Bertucci et al., 2019). Cells harboring a non-functional Rb protein have a dysfunctional proliferative capacity, continuing through the cell cycle unchecked even in the presence of CDK4/6 inhibitors. Combined analyses of ctDNA across three randomized trials of ET plus ribociclib therapy in patients with advanced breast cancer reported a low baseline incidence of *RB1* mutation in 1.7% of patients (Condorelli et al., 2018; O'Leary et al., 2018; Kumarasamy et al., 2022). Importantly, the addition of ribociclib to ET showed poor PFS in patients with tumors harboring RB1 mutations compared to wildtype (Kumarasamy et al., 2022). The low occurrence of RB1 mutation prior to use of CDK4/6 inhibitors compares with an incidence of 2%–9% in patients at the time of progression on CDK4/6 inhibitor therapy, suggesting that it is more commonly an acquired mechanism of resistance (Asghar et al., 2022). Loss of heterozygosity (LOH) of RB1 is also significantly associated with intrinsic CDK4/6 inhibitor resistance, and RB1 mutation is frequently acquired in CDK4/6 inhibitor treated patients and pre-clinical models with pre-existing RB1 LOH (Palafox et al., 2022; Safonov et al., 2022).

### 4.2 Aberrant cyclin E and CDK2 activity

The circumvention of CDK4/6 inhibition *via* the alternate phosphorylation of Rb through an upregulated CDK2-cyclin E axis represents another mechanism of CDK4/6 inhibitor resistance (Wang et al., 2007). Amplification of cyclin E1 and E2 results in an increase in CDK2 activity, and reduced expression of p27 (Taylor-Harding et al., 2015; Herrera-Abreu et al., 2016; Yang et al., 2017). There are however discordant results clinically. Higher levels of *CCNE1* mRNA were detected in resistant compared to sensitive tumors in the abemaciclib ABC-POP (Palafox et al., 2022) and palbociclib PALOMA-3 trials (Turner et al., 2019), whereas altered *CCNE1* expression was not significantly associated with an altered response to palbociclib plus letrozole in the PALOMA-2 trial (Finn et al., 2020).

*CDK2* mRNA expression has also been shown to be upregulated in palbociclib resistant ER + cell lines, and palbociclib sensitivity may be restored by *CDK2* knockdown with siRNA, resulting in tumor suppression, increased apoptosis, and senescence (Pandey et al., 2020). However, *CDK2* amplification has not been reported in clinical samples (Tadesse et al., 2020).

Aberrant CDK2 activation may also occur independently of CDK4/6 through mesenchymal-epithelial transition factor (c-MET) family receptor tyrosine kinase signaling and its downstream effector focal adhesion kinases (FAK) (Zhang et al., 2019). The incidence of acquired genomic aberrations in MET was approximately 8% in the ctDNA analysis of an abemaciclib monotherapy clinical trial (Goetz et al., 2020). Upregulation of the PI3K/AKT/mTOR pathway has also been shown to trigger non-canonical activity of CDK2 by binding to cyclin D, resulting in cell cycle progression and resistance to CDK4/6 inhibitors (Herrera-Abreu et al., 2016). These findings have led to interest in the clinical development of CDK2 inhibitors which will be described later.

### 4.3 Upregulation of CDK6 activity

Preclinical studies have demonstrated that CDK6 amplification may occur in response to prolonged CDK4/6 inhibitor exposure, and sensitivity can be restored following CDK6 knockdown (Alves et al., 2016; Yang et al., 2017). This finding was supported by the analysis of CDK6 protein expression in patients treated with combined CDK4/6 inhibitor and ET, whereby there was a significant inverse correlation with PFS (Al-Qasem et al., 2022). In the same study, the authors noted that a combined score of CDK6, p-CDK2, and cyclin E1 proteins also predicted a poorer outcome in this patient subgroup as well as in patients treated with ET alone.

Another mechanism resulting in increased CDK6 activity is through loss of *FAT1*, a member of the cadherin superfamily. Loss of function mutations in *FAT1* are present in about 2% of breast tumors (Li et al., 2018). Inactivation or deletion of *FAT1* results in activation of the Hippo signaling pathway which regulates cell growth and apoptosis (Steinhardt et al., 2008). Loss of FAT1 function results in an accumulation of YAP/TAZ transcription factors, which in turn promote overexpression of CDK6 and increase the growth inhibitory concentration of CDK4/6 inhibitors. This effect is most profound in biallelic FAT1 inactivation, where the PFS on CDK4/6 inhibitors have been reported at 2.4 months. In contrast, patients with missense mutations had only a slightly shorter PFS when compared to wildtype (10.1 vs. 11.3 months) (Li et al., 2018).

Exosomal miRNA-432-5p, a predicator target of SMAD4 and TGFBR3 can drive acquired CDK4/6 inhibitor resistance, independent of inherent genetic mutations, by increasing CDK6 expression, downregulation of SMAD4 and subsequent reduction in G_1_/S cell cycle arrest (Cornell et al., 2019). A “drug holiday” (removing palbociclib for a prolonged period) resulted in downregulation of CDK6, CCND1, and miRNA-432-5p while upregulating Rb. Consequently, the resistance of ER + breast cancer cells to palbociclib was reversed in *in vitro* and *in vivo* preclinical models (Cornell et al., 2019). This may explain why patients who have progressed on one CDK4/6 inhibitor may later respond to a different CDK4/6 inhibitor.

### 4.4 c-Myc alteration

C-Myc is activated by CDK2, 4 and 6, and has been shown to be upregulated in CDK4/6 inhibitor resistance in preclinical models (Pandey et al., 2020; Freeman-Cook et al., 2021). In support of this, ctDNA biomarkers analysis of the MONARCH-3 study demonstrated an enrichment of acquired *MYC* genomic alterations in the abemaciclib treatment arm (Goetz et al., 2020). c-Myc is also activated downstream of kinases such as S6K1. Elevated S6K1 have been reported in 9%–14% of breast cancer patients and studies have demonstrated that elevated S6K1 drives palbociclib resistance *via* activation of c-Myc signaling pathways in preclinical models and clinical breast cancer samples (Mo et al., 2022).

### 4.5 Activation of upstream signaling pathways

A study which compared whole exome sequencing data of CDK4/6 inhibitor resistant and sensitive breast tumors identified an enrichment of mutations and amplifications in *AKT1, KRAS, HRAS, NRAS, FGFR2* and *HER2* in the resistant samples (Wander et al., 2020), and similar findings were found in ctDNA biomarker analysis of the MONARCH-3 trial, whereby acquired mutations in *EGFR* and *FGFR1* were observed in the abemaciclib arm (Goetz et al., 2020). Interestingly, *PIK3CA* mutations were not more frequently found in the abemaciclib arm. Furthermore, high phospho-AKT levels in metastases have been shown to be a biomarker for poor prognosis and correlated with a shorter PFS in patients who have received CDK4/6 inhibitors and ET (Alves et al., 2021).

## 5 Therapeutic strategies following progression on CDK4/6 inhibitors

Candidate therapeutic strategies following progression on combination CDK4/6 inhibitor and ET can broadly be divided into 1) changing to an alternative ET and/or CDK4/6 inhibitor, 2) adding a therapy which inhibits a resistance mechanism of CDK4/6 inhibitors, such as inhibitors of CDK2 and PI3K/AKT/mTOR pathways, and 3) non-endocrine therapy approaches including chemotherapies and antibody drug conjugates (ADCs). Below, we summarize the clinical data to date for these strategies. Supplementary Table S1 and Supplementary Table S2 provide summaries of completed and ongoing clinical trials respectively in the post CDK4/6 inhibitor ER+/HER2-population.

### 5.1 Continuation of CDK4/6 inhibitors beyond progression

It is currently unclear if there is benefit from continued CDK4/6 inhibition post-progression on combination ET and CDK4/6 inhibitor. The phase II MAINTAIN trial enrolled patients with advanced ER+/HER2-breast cancer who progressed on ET and a CDK4/6 inhibitor, of whom 84% received prior palbociclib. Patients were randomized to receive a change in their ET backbone (exemestane or fulvestrant) plus ribociclib, versus ET (exemestane or fulvestrant) plus placebo. The median PFS was superior for the ET plus ribociclib arm compared to ET with placebo, supporting a switch in both ET and CDK4/6 inhibitor at progression (5.3 vs. 2.8 months; HR 0.56, *p* = 0.004) (Kalinsky et al., 2020; Kalinsky et al., 2022).

The phase II PACE trial similarly enrolled patients with advanced ER+/HER2-breast cancer who received prior AI and CDK4/6 inhibitor, of which 91% received palbociclib (Mayer et al., 2022). Patients were randomized to fulvestrant alone, fulvestrant plus palbociclib, or fulvestrant plus palbociclib plus avelumab, a PD-L1 inhibitor. In contrast to the MAINTAIN study, there was no significant difference in PFS for the fulvestrant plus palbociclib arm compared to the fulvestrant alone arm (4.6 vs. 4.8 months respectively), suggesting that a switch in both ET and CDK4/6 inhibitor may be required to derive additional benefit. There interestingly was a longer PFS noted in patients receiving avelumab (8.1 months) which may warrant further investigation (Mayer et al., 2022). The triplet combination of atezolizumab, abemaciclib and fulvestrant is one of the treatment regimens being assessed in the ongoing MORPHEUS HR + BC (Clinicaltrials.gov identifier NCT03280563) platform trial.

The phase II BioPER single-arm study enrolled 33 patients with advanced ER+/HER2-breast cancer who had prior clinical benefit to but subsequently progressed on palbociclib plus ET (Albanell et al., 2023). Patients continued palbociclib but had switch of their ET. Clinical benefit rate was 34% and median PFS was 2.6 months (Albanell et al., 2023). A composite biomarker signature incorporating low Rb score (defined as <1% tumor cells with positive nuclear staining on immunohistochemistry), high cyclin E1 (defined as ≥10% tumor cells with positive nuclear staining on immunohistochemistry), and *ESR1* mutation was associated with poorer PFS (Albanell et al., 2023). Other trials evaluating continued CDK4/6 inhibition are in progress and included in Supplementary Table S2.

Additional insights can be gained from a retrospective review of 87 patients who received abemaciclib with or without ET, following progression on palbociclib with ET, where the median PFS and OS were 5.3 and 17.2 months respectively (Wander et al., 2021). These results were remarkably similar to the MONARCH 1 study, where patients with ER+/HER2-breast cancer received abemaciclib following prior ET and chemotherapy but were CDK4/6 inhibitor naïve (Dickler et al., 2017). Interestingly, PFS was longer for patients who received sequential CDK4/6 inhibitor, compared to patients who had intervening non-CDK4/6 inhibitor therapy, even after adjusting for number of prior lines of therapy (Wander et al., 2021).

### 5.2 Novel endocrine therapies

There are several emerging novel classes of ET, of which the oral SERDs are the largest group and are reviewed in detail elsewhere (Downton et al., 2022). To date there have been four completed randomized trials of oral SERDs in advanced ER+/HER2-breast cancer, after progression on prior ET with or without a CDK4/6 inhibitor, and results have been mixed. In both the phase III EMERALD trial of elacestrant *versus* physician’s choice ET (Bidard et al., 2022) and the phase II SERENA-2 trial of camizestrant *versus* fulvestrant (Oliveira et al., 2022), oral SERD treatment was associated with a statistically significant PFS benefit. In contrast, the phase II AMEERA-3 (Tolaney et al., 2022) and the phase II acelERA trial (Martin Jimenez et al., 2022) of amcenestrant and giredestrant respectively were both negative trials with no significant difference in PFS observed between the oral SERDs and physician’s choice ET. Of note, these four trials were of oral SERD monotherapy, EMERALD was the only trial that mandated prior CDK4/6 inhibitor, and there was heterogeneity across trials in the proportion of patients with *ESR1* mutant disease. The *ESR1* mutant subgroup derived a greater benefit compared to the *ESR1* wildtype subgroups across these trials, supporting its use as a biomarker to identify ongoing ER dependence in this pretreated population of patients. Elacestrant is the first oral SERD to be FDA approved following progression on first line ET and CDK4/6 inhibitors in January 2023.

Other novel ET agents in development include the proteolysis targeting chimera (PROTAC) ARV-471 (Hurvitz et al., 2022), the complete ER antagonist (CERAN) OP-1250 (Patel et al., 2022), the selective ER covalent antagonists (SERCA) H3B 6545 (Johnston et al., 2022), the third-generation selective ER modulator (SERM) lasofoxifene (Goetz et al., 2022a; Damodaran et al., 2022), and the ShERPA (selective human ER partial agonists) TTC-352 (Dudek et al., 2020). These agents have demonstrated encouraging anti-tumor activity in early phase trials which have included patients treated previously with a CDK4/6 inhibitor (Supplementary Table S1).

Finally, there is preclinical data to support AR activation as a therapeutic strategy for ER + breast cancer, including ET and CDK4/6 inhibitor resistant ER + breast cancer models (Hickey et al., 2021). AR activation results in an altered distribution of ER chromatin binding and its interaction with essential co-activators (p300, SRC-3), resulting in the repression of ER-regulated cell cycle genes (Panet-Raymond et al., 2000). This has prompted clinical trials evaluating the selective androgen receptor modulator (SARM) enobosarm as monotherapy (Clinicaltrials.gov identifier NCT04869943) and in combination with abemaciclib (Clinicaltrials.gov identifier NCT05065411) in patients who have progressed on ET and a CDK4/6 inhibitor.

### 5.3 CDK2 inhibitor combinations

In light of the evidence for the role of CDK2 and cyclin E in CDK4/6 inhibitor resistance, another therapeutic approach would be to target CDK2 either concurrently with or following the development of CDK4/6 inhibitor resistance (Tadesse et al., 2020). Preclinical studies have demonstrated increased efficacy using the non-selective CDK2 inhibitor dinaciclib concurrently with palbociclib and letrozole, compared with palbociclib and letrozole alone (Al-Qasem et al., 2022). More specific CDK2 inhibitors are currently under evaluation in early phase trials enrolling patients with solid tumors including ER+/HER2-breast cancer. These include PF-07104091 (Clinicaltrials.gov identifiers NCT04553133 and NCT05262400), BLU-222 (Clinicaltrials.gov identifier NCT05252416), and the CDK2/4/6 inhibitor PF-06873600 (Clinicaltrials.gov identifier NCT03519178).

### 5.4 Combination therapies that target the PI3K/AKT/mTOR pathway

Similar to CDK4/6 inhibitors, small molecule inhibitors of the PI3K/AKT/mTOR pathway are also FDA approved for use in endocrine resistant ER+/HER2-breast cancer in combination with ET. Upregulation of this pathway is associated with ET and CDK4/6 inhibitor resistance as described earlier. Given the potential benefit of continued CDK4/6 inhibition and known crosstalk between the CDK4/6 and PI3K/AKT/mTOR pathways, a number of combinations that include inhibitors of PI3K/AKT/mTOR are under evaluation in patients who have progressed on CDK4/6 inhibitors. In addition to therapeutic efficacy, key considerations include toxicities and the costs of these multidrug regimens.

The seminal phase III SOLAR-1 trial compared alpelisib plus fulvestrant *versus* placebo plus fulvestrant in patients with advanced ER+/HER2-breast cancer who had relapsed after or progressed on prior AI. OS was extended by 7.9 months in patients with *PIK3CA* mutated cancer, establishing *PIK3CA* mutation as a predictive biomarker for alpelisib benefit (Andre et al., 2021). Notably, only 6% of participants in SOLAR-1 received a prior CDK4/6 inhibitor (Andre et al., 2019). In contrast, the phase II BYLieve trial assessed alpelisib and fulvestrant following progression on CDK4/6 inhibitors in patients with a detectable *PIK3CA* mutation. The median PFS and OS was lower than those observed in the *PIK3CA* mutated cohort of SOLAR-1, suggesting that the benefit of alpelisib may be attenuated in the setting of CDK4/6 inhibitor resistance (Rugo et al., 2021). Other studies of alpelisib and fulvestrant following progression on CDK4/6 inhibitors are ongoing and include CAPTURE (Australian Trials identifier ACTRN12619001117101), EPIK-B5 (Clinicaltrials.gov identifier NCT05038735), and SEQUEL-Breast (NCT05392608).

*In vitro* studies and analysis of patient samples demonstrate that activating *AKT* aberrations are enriched following treatment with CDK4/6 inhibitors and are associated with CDK4/6 inhibitor resistance, which contrasts with PI3K alterations (Wander et al., 2020). The combination of fulvestrant, palbociclib and capiversertib (an inhibitor of AKT1, AKT2 and AKT3) has been shown to be effective in suppressing tumor growth in preclinical models that were dually resistant to ET and CDK4/6 inhibitors (Alves et al., 2021). In the phase II FAKTION trial, patients with endocrine-resistant advanced ER+/HER2-breast cancer were randomized to receive fulvestrant and capiversertib versus fulvestrant and placebo (Jones et al., 2020). This trial included only CDK4/6 inhibitor naïve patients. PFS and OS were superior in the capiversertib arm, and the benefits were more pronounced in patients with evidence of PI3K/AKT/PTEN pathway alterations (Howell et al., 2022). Extending on this study, the phase III CAPItello-291 trial evaluated the same treatments and included 69% patients who had received prior CDK4/6 inhibitor therapy, and reported a significant improvement in PFS in the arm containing capiversertib compared with placebo (7.2 vs. 3.6 months; HR 0.60, *p* < 0.001) (Turner et al., 2022). The FINER trial (Clinicaltrials.gov identifier NCT04650581) is currently in progress assessing the combination of another AKT inhibitor ipatasertib with fulvestrant, after progression on ET and CDK4/6 inhibitors.

Data for everolimus (mTOR inhibitor) plus exemestane after progression on CDK4/6 inhibitors is limited to two small real-world studies. In these studies, median time on treatment or PFS was limited to a few months (Lupichuk et al., 2019; Cook et al., 2021). The phase I/II TRINITI trial evaluated the triplet combination of exemestane, ribociclib, and everolimus, of whom 92% had received prior CDK4/6 inhibitors. The clinical benefit rate (CBR) at week 24 was 41%, and median PFS was 5.7 months, an impressive result considering the heavily pretreated group of patients (Bardia et al., 2021). The phase Ib B2151009 trial assessed the pan-PI3K/mTOR inhibitor gedatolisib in combination with palbociclib and ET. Patients had received 0–3 prior lines of therapy for metastatic ER+/HER2-breast cancer, and 12-month PFS in this trial was 53% in patients who had received prior CDK4/6 inhibitor therapy (Wesolowski et al., 2022). CAPItello-292 (Clinicaltrials.gov identifier NCT04862663) and VIKTORIA-1 (Clinicaltrials.gov identifier NCT05501886) are other ongoing trials of triplet endocrine, CDK4/6 inhibitor and PI3K/AKT/mTOR pathway inhibitor therapy.

### 5.5 Non-endocrine therapy approaches

Chemotherapy remains an option for patients who have progressed on ËT alone and in combination with CDK4/6 inhibitors. It is generally held in reserve for rapidly progressive or endocrine-refractory disease. The development of new classes of ET is aimed at delaying the switch to chemotherapy, but such a strategy is only effective in patients whose tumors retain some dependence on ER signaling.

An emerging class of therapies used in ER + breast cancer is antibody drug conjugates (ADCs). These are complex molecules consisting of tumor-antigen targeting antibodies linked to a potent chemotherapy payload and have immune-mediated and cytotoxic properties. In addition to direct cytotoxic effects, ADCs with a cleavable linker can also exert bystander killing effects on neighboring cells which may or may not express the target antigen (Staudacher and Brown, 2017).

Trastuzumab deruxtecan is a HER2-directed ADC, consisting of trastuzumab, a humanized anti-HER2 antibody linked to a topoisomerase I inhibitor payload, approved for use in advanced HER2-positive breast cancer (Cortes et al., 2022). It was more recently evaluated in and demonstrated benefit also against tumors with HER2-low expression (defined as immunohistochemistry 1 + or 2+ with a negative *in situ* hybridization result) (Modi et al., 2020). In the pivotal phase III DESTINY-Breast04 trial, patients were randomized to receive trastuzumab deruxtecan or physician’s choice chemotherapy. 89% of patients had ER + disease, and of which, 70% had received prior CDK4/6 inhibitor therapy. In the ER + subgroup, the trastuzumab deruxtecan arm had an improved PFS (10.1 vs. 5.4 months; HR 0.51, *p* < 0.001) and OS (23.9 vs. 17.5 months; HR 0.64, *p* = 0.003) compared to the treatment of physician’s choice arm, and the benefit was similar for those who had received and not received prior CDK4/6 inhibitors (Modi et al., 2022).

Sacituzumab govitecan is an ADC, which is directed against trophoblast cell-surface antigen 2 (Trop-2) and is linked to a topoisomerase I inhibitor SN-38, cytotoxic payload (Syed, 2020). Trop-2 is a transmembrane glycoprotein which is overexpressed in many epithelial tumors, including >90% of ER+/HER2-breast cancers (Vidula et al., 2022). In the phase III TROPiCS-02 trial, patients with heavily pretreated (median of three prior systemic therapies) ER+/HER2-breast cancer and who received prior CDK4/6 inhibitors were randomized to sacituzumab govitecan or physician’s choice chemotherapy. The median PFS was 5.5 months for sacituzumab govitecan *versus* 4.0 months for chemotherapy (HR 0.66; *p* = 0.0003), and benefit was independent of Trop-2 tumor status (Rugo H. et al., 2022; Rugo et al., 2022b; Rugo et al., 2022c). The outcomes of DESTINY-Breast04 and TROPiCS-02 have established a clinical role for ADCs following progression on CDK4/6 inhibitors.

A third ADC datopotamab deruxtecan, which also acts against Trop-2 and uses the same payload as trastuzumab deruxtecan has had encouraging results in a phase I trial that included heavily pretreated ER+/HER2-breast cancer (Meric-Bernstam et al., 2022), and is under further assessment in the phase III TROPION-Breast-01 trial (Clinicaltrials.gov identifier NCT05104866).

## 6 Conclusion

The combination of ET with a CDK4/6 inhibitor is the standard of care first line treatment for advanced ER+/HER2-breast cancer, and while practice changing and improving survival outcomes, patients eventually develop progressive disease due to intrinsic or acquired drug resistance. There are various systemic treatment options post-progression, but it is not currently clear how these are best sequenced. This represents a current clinical knowledge gap, and defining a biomarker led approach in this setting is critical. Patients whose tumors retain dependence of ER signaling can benefit from changing the endocrine therapy backbone, and where the mechanisms of resistance to combination ET and CDK4/6 inhibitor can be identified and targeted, this may represent a rationale strategy to delay the start of chemotherapy.

Current targeted therapy options include PI3K and PARP inhibitors in the setting of *PIK3CA* and *BRCA1/2* mutations respectively (Burstein et al., 2021), but their use is limited by lack of universal access to genomic testing and treatment side effect profiles. There is an unmet need for efficacious and better tolerated therapies and this review has discussed the preclinical backdrop and status of continued CDK4/6 inhibition, addition of CDK2 inhibition, newer ET backbones, PI3K/AKT/mTOR inhibitors, AR agonists, and ADCs as strategies in the post-CDK4/6 inhibitor ER+/HER2-treatment landscape. The novel approach delivering chemotherapy to tumors through ADCs represent an exciting strategy which has been shown to be effective. The novel approach of delivering chemotherapy to tumors through ADCs represent an exciting strategy which has been shown to be effective.

There is intra- and inter-patient heterogeneity in breast cancer tumor biology, mechanisms of drug resistance, and tumor genomic profiles. Identification of biomarkers relevant to both intrinsic and acquired resistance may inform how to personalize the treatment approach for ER+/HER2-breast cancer patients. The major challenge is to accrue patients of each unique resistance subset for future clinical trials to test new combinations in the post-combination ET plus CDK4/6 inhibitor setting.
