# Supplementary material for: CDK4/6 inhibitor resistance in estrogen receptor positive breast cancer, a 2023 perspective
Source: Front Cell Dev Biol. 2023 Mar 22;11:1148792. doi: 10.3389/fcell.2023.1148792 (PMC10073728; doi:10.3389/fcell.2023.1148792)
Supplement: Supplementary file 1 [file DataSheet1.docx]

Supplementary Table 1. Completed trials of therapies post-CDK4/6 inhibitor (CDK4/6i) in advanced ER+/HER2- breast cancer.

| **Trial name and NCT identifier** | **Phase** | **N** | **Population and prior therapies** | **Median (range) prior lines** | **% Prior CDK4/6i** | **Median (range) prior lines chemotherapy metastatic setting** | **Investigational Arm** | **Comparator Arm** | **Randomization** | **ORR** | **CBR** | **Median PFS** | **Median OS** |
| --- | --- | --- | --- | --- | --- | --- | --- | --- | --- | --- | --- | --- | --- |
| Continuation of CDK4/6i beyond progression | | | | | | | | | | | | | |
| MAINTAIN (Kalinsky et al., 2022).  NCT02632045 | II | 119 | Prior ET + any CDK4/6i, ≤1 line prior chemotherapy | NR | 100% | 0 (0-1)  9% prior chemotherapy | Ribociclib + ET (Fulvestrant or exemestane) | Placebo + ET (Fulvestrant or exemestane) | Open-label randomized 1:1, 2-arm | 20% vs 11% | 43% vs 25% | 5.3 months vs 2.8 months | NR |
| PACE (Mayer et al., 2022)  NCT03147287 | II | 220 | Prior ET 1-2 lines, prior CDK4/6i in adjuvant or metastatic setting (not necessarily most recent line), ≤1 line prior chemotherapy, no prior SERD | 1 (0-2+) | 100% | 0 (0-1)  16% prior chemotherapy | Fulvestrant + Palbociclib;  Fulvestrant + Palbociclib + Avelumab | Fulvestrant monotherapy | Open-label randomized 1(F):2(F+P):1(F+P+A), 3-arm | 11% (F) vs 14% (F+P) vs 18% (F+P+A) | 19% (F) vs 32% (F+P) vs 35% (F+P+A) | 4.8 months (F) vs 4.6 months (F+P) vs 8.1 months (F+P+A) | 27.5 months (F) vs 24.6 months (F+P) vs 42.5 months (F+P+A) |
| BioPAR (Albanell et al., 2023)  NCT03184090 | II | 33 | Prior ET 1-2 lines, prior Palbociclib, ≤1 line prior chemotherapy | 2 (1-4) | 100% | 0 (0-1)  13% prior chemotherapy | Palbociclib + Physician’s choice ET | N/A | Non-randomized, single-arm | 6% | 34% | 2.6 months | 23.9 months |
| Novel ET backbones | | | | | | | | | | | | | |
| EMERALD (Bidard et al., 2022)  NCT03778931 | III | 477 | Prior ET 1-2 lines, prior CDK4/6i, ≤1 line prior chemotherapy | 1 (1-2) ET | 100% | 0 (0-1)  22% prior chemotherapy | Elacestrant (oral SERD) | Physician’s choice ET | Open-label, randomized 1:1, 2-arm | NR | NR | 12 month PFS 22.3% vs 9.4% | Not mature |
| SERENA-2 (Oliveira et al., 2022)  NCT04214288 | II | 240 | Prior ET (≤1 line in advanced setting), ≤1 line prior chemotherapy, no prior SERD | 1 (0-1) | 50% | 0 (0-1)  19% prior chemotherapy | Camizestrant (oral SERD) at 3 dose levels (75mg, 150mg, 300mg) | Fulvestrant | Open-label, randomized 1:1:1:1, 4-arm | 16-20% vs 12% | 49-51% vs 39% | 7.2 months (75mg) and 7.7 months (150mg) vs 3.7 months (75mg) and 3.7 months (150mg) | Not mature |
| AMEERA-3 (Tolaney et al., 2022)  NCT04059484 | II | 290 | Prior ET 1-2 lines, prior CDK4/6i allowed but not mandatory, ≤1 line prior chemotherapy or targeted agent | 1 (0-2) ET | 79% | 0 (0-1)  11% prior chemotherapy | Amcenestrant (oral SERD) | Physician’s choice ET | Open-label, randomized 1:1, 2-arm | NR | NR | 3.6 months vs 3.7 months | Not mature |
| acelERA trial (Martin Jimenez et al., 2022)  NCT04576455 | II | 303 | 1-2 lines prior systemic therapy (≤1 line chemotherapy, ≤1 line targeted therapy) | 1 (1-2) | 42% | 0 (0-1)  32% prior chemotherapy | Giredestrant (oral SERD) | Physician’s choice ET | Open-label, randomized 1:1, 2-arm | 13% vs 7% | 32% vs 21% | 5.6 months vs 5.4 months | Not mature |
| (Hurvitz et al., 2022)  NCT04072952 | II | 71 | ≥1 prior ET for ≥6 months, prior CDK4/6i, ≤1 line prior chemotherapy | 4 (1-10) | 100% | 0 (0-1)  45% prior chemotherapy | ARV-471 (PROTAC) | N/A | Dose expansion (post phase I dose escalation) at 2 dose levels | NR | 38% | 3.5 months (all)  5.5 months (*ESR1* mutant) | NR |
| (Patel et al., 2022)  NCT04505826 | I/II | 41 | Prior ET | 3 (range NR) | 95% | NR  42% prior chemotherapy | OP-1250 (CERAN) | N/A | Dose escalation and expansion | 8% | 29% | NR | NR |
| (Hamilton et al., 2022)  NCT03250676 | I/II | 94 | Prior ET unlimited, ≤1 line prior chemotherapy | 3 (1-8) | 87% | NR  50% prior chemotherapy | H3B 6545 (SERCA) | N/A | Dose escalation and expansion | 17% | 40% | 5.1 months | NR |
| (Johnston et al., 2022)  NCT04288089 | Ib | 22 | Dose-expansion: ≥2 prior ET, ≤1 line prior chemotherapy  Dose-expansion: ≤2 prior ET and ≤1 line prior chemotherapy, but no CDK4/6i | 3 (1-6) | 77% | 0 (0-1)  36% prior chemotherapy | H3B 6545 + Palbociclib | N/A | Dose escalation and expansion | 18% | 55% | NR | NR |
| ELAINE-1 (Goetz et al., 2022)  NCT03781063 | II | 103 | Prior ET + CDK4/6i, ≤1 line prior chemotherapy, *ESR1* mutant | NR | 100% | 0 (0-1)  6% prior chemotherapy | Lasofoxifene (3^rd^ generation SERM) | Fulvestrant | Open-label, randomized 1:1, 2-arm | 13% vs 3% | 37% vs 22% | 6.0 months vs 4.0 months | NR |
| ELAINE-2 (Damodaran et al., 2022)  NCT04432454 | II | 29 | Prior ET 1-2 lines, prior CDK4/6i allowed but not mandatory, ≤1 line prior chemotherapy, *ESR1* mutant | NR | 97% | 0 (0-1)  48% prior chemotherapy | Lasofoxifene + Abemaciclib | N/A | Single-arm | 33% | 62% | 13.9 months | NR |
| (Dudek et al., 2020)  NCT03201913 | I | 15 | Prior ET ≥2 lines, prior CDK4/6i | NR | 100% | 0 (0-1)  7% prior chemotherapy | TTC-352 (ShERPA) | N/A | Dose escalation and expansion | 0% | 40% | 1.9 months | NR |
| Combination therapies that target the PI3K/AKT/mTOR pathway | | | | | | | | | | | | | |
| BYLieve cohort A (Rugo et al., 2021)  NCT03056755 | II | 127 cohort A | Prior AI and CDK4/6i (CDK4/6i must be last regimen prior to study entry)  ≤2 prior lines in metastatic setting, including ≤1 line prior chemotherapy, *PIK3CA* mutant | 1 (0-3) | 100% | NR | Alpelisib + Fulvestrant | N/A | Non-randomized, non-comparative, 3-cohort | 17% | 45% | 7.3 months | 17.3 months |
| PIKNIK (Savas et al., 2022)  NCT02506556 | II | 33 (ER+/HER2- cohort) | ≥1 prior anti-cancer therapy in metastatic setting, somatic PI3K aberration | 3 (1-9) | 24% | NR | Alpelisib | N/A | Single-arm | 30% | 36% | 5.4 months | NR |
| TAKTIC (Wander et al., 2022)  NCT03959891 | Ib | 77 | Prior ET + CDK4/6i, ≤2 lines prior chemotherapy  Arm A: Ipatasertib + Fulvestrant  Arm B: Ipatasertib + AI  Arm C: Ipatasertib + Fulvestrant + Palbociclib | 3 (1-13) | 84% | NR | Ipatasertib (AKT inhibitor) + ET (Fulvestrant or AI) | N/A | Non-randomized, 3-cohort. Arm C included dose escalation phase | 20% | Arm A: 53%  Arm B: 31%  Arm C: 57% | Arm A: 5.5 months | Arm A: 24.5 months |
| CAPItello-291 (Turner et al., 2022)  NCT04305496 | III | 708 | Prior AI (may be in adjuvant or metastatic setting)  ≤2 prior ET and ≤1 line prior chemotherapy in metastatic setting, *PIK3CA* mutant | 1 (0-2) ET | 69% | 0 (0-1)  18% prior chemotherapy | Capivasertib + Fulvestrant | Placebo + Fulvestrant | Double-blind, randomized 1:1, 2-arm | 23% vs 12% (all)  29% vs 10% (AKT-altered subgroup) | 73% vs 54% (all)  79% vs 50% (AKT-altered subgroup) | 7.2 months vs 3.6 months (all)  7.3 months vs 3.1 months (AKT-altered subgroup) | Not mature |
| TRINITI-1 (Bardia et al., 2021)  NCT02732119 | I/II | 104  (25 phase I, 79 phase II) | 1-3 prior lines ET and ≤1 chemotherapy  II: must have progressed after ≥4 months of CDK4/6i as most recent line | 1 (1-8) | 100% | 0 (0-1)    13% prior chemotherapy | Exemestane + Ribociclib + Everolimus | Different doses of Ribociclib and Everolimus within the combination assessed within phase I | I: dose escalation and de-escalation, 3 cohorts  II: dose expansion, single arm | 8% | 41% | 5.4 months | NR |
| B2151009 (Wesolowski et al., 2022)  NCT02684032 | Ib | 141 | Prior CDK4/6i not mandatory; ≤1 prior chemotherapy  Arm A: First-line  Arm B: Second or later line, CDK4/6i naïve  Arm C: Second or third line, CDK4/6i pretreated; gedatolisib weekly dosing  Arm D: Second or third line, CDK4/6 pretreated; gedatolisib intermittent dosing | 0-2 depending on enrolled arm | 100% in arms C and D | NR | ET (Letrozole or Fulvestrant) + Gedatolisib (dual PI3K/mTOR inhibitor) + Palbociclib | N/A | Dose escalation and 4-arm expansion | Arm A: 81%  Arm B: 77%  Arm C: 36%  Arm D: 63% | NR | Arm A: not reached  Arm B: 12.9 months  Arm C: 5.1 months  Arm D: 12.9 months | NR |
| Antibody Drug Conjugates | | | | | | | | | | | | | |
| DS-8201a in Solid Tumors (Modi et al., 2020)  NCT02564900 | Ib | 47 ER+ cohort (54 all patients) | HER2-low, refractory to standard treatments | 7 (2-16) all patients | 30% all patients | NR | Trastuzumab deruxtecan | N/A | Single-arm | 37% (all) | 87% (all) | 11.1 months (all) | 29.4 months (all) |
| DESTINY-Breast04 (Modi et al., 2022)  NCT03734029 | III | 494 ER+ cohort | HER2-low, ≥1 prior ET and 1-2 lines of chemotherapy | 3 (1-9) | 70% | NR | Trastuzumab deruxtecan | Physician’s choice chemotherapy (Capecitabine, Paclitaxel, Nab-paclitaxel, Gemcitabine, Eribulin) | Open-label, randomized 2:1, 2-arm | 53% vs 16% | 71% vs 34% | 10.1 months vs 5.4 months | 23.9 months vs 17.5 months |
| IMMU-132-01 (Kalinsky et al., 2020)  NCT01631552 | I/II | 54 | ≥1 prior ET, ≥1 line prior chemotherapy | NR | 61% | NR | Sacituzumab govitecan | N/A | Single-arm | 31% (all)  25% (prior CDK4/6i) | 44% (all)  38% (prior CDK4/6i) | 5.5 months (all)  3.8 months (prior CDK4/6i) | 12 months (all)  11 months (prior CDK4/6i) |
| TROPiCS-02 (Rugo et al., 2022a; Rugo et al., 2022b)  NCT03901339 | III | 543 | ≥1 prior ET, prior taxane, prior CDK4/6i in any setting; 2-4 lines prior chemotherapy in metastatic setting | 7 (3-17) | 99% | 3 (0-8) | Sacituzumab govitecan | Physician’s choice chemotherapy (Capecitabine, Vinorelbine, Gemcitabine, Eribulin) | 1:1 | 21% vs 14% | 34% vs 22% | 5.5 months vs 4.0 months | 14.4 months vs 11.2 months; Not yet mature |
| TROPION-PanTumor01 (Meric-Bernstam et al., 2022)  NCT03401385 | I | 41 | Advanced solid tumors including ER+/HER2- breast cancer. ER+/HER2- participants had prior ET and 1-3 lines prior chemotherapy | 5 (3-10) | 95% | NR | Datopotamab Deruxtecan | N/A | Dose-escalation and expansion | 29% | 41% | NR | NR |

Abbreviations: CBR = clinical benefit rate, CDK4/6i = CDK4/6 inhibitor, ER = estrogen receptor, ET = endocrine therapy, N = total patient enrolment; N/A = not applicable, NR = not reported, ORR = objective response rate, OS = overall survival, PFS = progression free survival, SERD = selective estrogen receptor degrader.

Supplementary Table 2. Ongoing trials of therapies post-CDK4/6 inhibitor (CDK4/6i) in advanced ER+/HER2- breast cancer.

| **Trial Name and NCT identifier** | **Phase** | **N** | **Patient population** | **Design and randomization** | **Arms** | **Primary endpoint** |
| --- | --- | --- | --- | --- | --- | --- |
| Continuation of CDK4/6i beyond progression | | | | | | |
| EMBER-3  NCT04975308 | III | 800* | Prior AI and CDK4/6i (Palbociclib or Ribociclib), no prior chemotherapy in advanced setting, no prior SERD nor PI3K/AKT/mTOR inhibitor | Open-label randomized 1:1:1, 3-arm | Imlunestrant (oral SERD) monotherapy  Vs Imlunestrant + Abemaciclib  Vs Physician’s choice ET (Fulvestrant or Exemestane) | PFS in all comers and in *ESR1* mutant population |
| PALMIRA  NCT03809988 | II | 198* | Prior ET + Palbociclib in adjuvant or first-line metastatic setting | Open-label, randomized 1:1, 2-arm | New ET partner (Letrozole or Fulvestrant) + continued Palbociclib  Vs New ET partner (Letrozole or Fulvestrant) monotherapy | PFS |
| postMONARCH  NCT05169567 | III | 350* | Prior ET and CDK4/6i in adjuvant or first line metastatic setting; ≤1 line of therapy for advanced disease, no prior chemotherapy for advanced disease; no prior SERD nor PI3K/AKT/mTOR inhibitor | Double-blind randomized 1:1, 2-arm | Abemaciclib + Fulvestrant  Vs Placebo + Fulvestrant | PFS |
| Combination of CDK4/6i with checkpoint inhibitors | | | | | | |
| MORPHEUS HR+BC  NCT03280563 | Ib/II | 138 | Progression on CDK4/6i in first or second-line setting | Umbrella trial with 2 sequential stages; Randomized in stage 1 | Stage I: Fulvestrant vs Atezolizumab-containing doublet or triplet combination (combinations include Atezolizumab + Abemaciclib + Fulvestrant)  Stage 2: Different triplet combination | ORR |
| CDK2 inhibition | | | | | | |
| NCT03519178 | I/IIa | 155 | Advanced solid tumors including ER+/HER2- breast cancer; prior ET + CDK4/6i and 1-2 lines of prior chemotherapy | Dose-escalation then expansion, sequential multi-arm, non-comparative | PF-06873600 (CDK2/4/6 inhibitor)  PF-06873600 + Letrozole  PF-06873600 + Fulvestrant | Safety and tolerability, ORR |
| VELA  NCT05252416 | I/II | 366* (all tumor subtypes included) | Advanced/relapsed solid tumors including ER+/HER2- breast cancer that has progressed on prior CDK4/6i | Dose-escalation then expansion, sequential multi-arm, non-comparative | BLU-222 (CDK2 inhibitor) monotherapy  BLU-222 + Carboplatin  BLU-222 + Ribociclib + Fulvestrant  BLU-222 + Fulvestrant | Maximum Tolerated Dose, Recommended Phase 2 Dose, Adverse Events, ORR |
| C4161001  NCT04553133 | I/IIa | 240* (all tumor subtypes) | Advanced solid tumors including ER+/HER2- breast cancer; ≥2 prior lines in advanced setting including ET + CDK4/6i and ≥2 chemotherapy | Dose-escalation then expansion, sequential multi-arm, non-comparative | PF-07104091 (CDK2 inhibitor) monotherapy  PF-07104091 + Palbociclib + Fulvestrant  PF-07104091 + Palbociclib + Letrozole | Adverse events, ORR |
| NCT05262400 | Ib/II | 144* (all tumor subtypes) | Advanced solid tumors including ER+/HER2- breast cancer; ≥1 prior line in advanced setting including ET + CDK4/6i | Dose-escalation then expansion, sequential multi-arm, non-comparative | PF-07220060 (CDK4 inhibitor) + PF-07104091 (CDK2 inhibitor) – dose-escalation  PF-07220060 + PF-07104091 + Fulvestrant – dose-expansion  PF-07220060 + PF-07104091 + Letrozole – dose-expansion | Adverse events |
| Novel ET backbones | | | | | | |
| VERITAC-2  NCT05654623 | III | 560* | Prior ET 2 lines, prior CDK4/6i, no prior chemotherapy for advanced disease, no prior fulvestrant | Open-label, randomized 1:1, 2-arm | ARV-471 (PROTAC)  Vs Fulvestrant | PFS |
| NCT05501769 | Ib | 32* | 1-3 prior lines in advanced setting including ≥1 ET, prior CDK4/6i, and ≤1 prior chemotherapy | Dose escalation and expansion | ARV-471 + Everolimus | Adverse events, Recommended Phase 2 Dose |
| TACTIVE-U  NCT05573555 | Ib/II | 35 | ≤2 prior lines in advanced setting; must have had prior CDK4/6i (in adjuvant or metastatic setting) | Dose escalation and expansion | ARV-471 + Ribociclib | Adverse events, ORR |
| NCT04072952 | Ib/II | 215 | ≥1 prior ET, prior CDK4/6i and 0-3 prior chemotherapy (varies for different parts of trial) | Dose escalation and expansion | ARV-471  ARV-471 + Palbociclib | Adverse events |
| NCT04505826 | I/II | 94 | Prior ET | Dose escalation and expansion | OP-1250 (CERAN) | Maximum Tolerated Dose, Recommended Phase 2 Dose |
| NCT05508906 | Ib | 60 | ≤2 prior lines ET and ≤1 prior chemotherapy | Dose escalation and expansion, 2-groups, non-comparative | OP-1250 + Ribociclib  OP-1250 + Alpelisib | Adverse events, Maximum Tolerated Dose, Recommended Phase 2 Dose |
| NCT05266105 | Ib | 30 | Prior ET | Dose escalation and expansion | OP-1250 + Palbociclib | Dose Limiting Toxicities, Adverse events |
| Androgen Receptor Agonists | | | | | | |
| ENABLAR-2  NCT05065411 | III | 186* | Prior ET + Palbociclib; androgen receptor nuclei staining ≥40% | Open-label randomized 1:1, 2-arm | Enobosarm + Abemaciclib  Vs Non-steroidal AI, Exemestane +/- Everolimus, or fulvestrant | PFS |
| ARTEST  NCT04869943 | III | 210* | ≥ 2 prior lines in metastatic setting including Non-steroidal AI, Fulvestrant, and CDK4/6i; ≤1 chemotherapy in metastatic setting; androgen receptor nuclei staining ≥40% | Open-label, randomized 1:1, 2-arm | Enobosarm  Vs Exemestane monotherapy, Exemestane + Everolimus, or SERM | PFS |
| Combination therapies that target the PI3K/AKT/mTOR pathway | | | | | | |
| CAPTURE  ACTRN12619001117101 | II | 140* | *PIK3CA* mutation present in ctDNA, prior AI and CDK4/6i (may be in (neo) adjuvant or metastatic setting), ≥2 prior ET and ≥1 chemo in metastatic setting | Open label, randomized 1:1, 2-arm | Alpelisib + Fulvestrant  Vs Capecitabine | PFS |
| EPIK-B5  NCT05038735 | III | 234* | *PIK3CA* mutation present in tissue, prior AI and CDK4/6i (may be in adjuvant or metastatic setting), ≥1 chemotherapy in metastatic setting, no prior fulvestrant or PI3K/mTOR/AKT inhibitor | Double-blind randomized 1:1, 2-arm | Alpelisib + Fulvestrant  Vs Placebo + Fulvestrant | PFS |
| SEQUEL-Breast  NCT05392608 | II | 130* | *PIK3CA* mutation present, prior CDK4/6i in first or second line advanced setting, fulvestrant in latest line, no prior PI3K/mTOR/AKT inhibitor | Single-arm | Alpelisib + Fulvestrant (fulvestrant continued beyond progression) | PFS |
| FINER  NCT04650581 | III | 250* | Prior 1^st^-line AI + CDK4/6i as latest line, no prior fulvestrant or SERD or PI3K/mTOR/AKT inhibitor | Double-blind randomized 1:1, 2-arm | Ipatasertib + Fulvestrant  Vs Placebo + Fulvestrant | PFS |
| CAPItello-292  NCT04862663 | Ib/III | 700 (72 phase Ib, 628 phase III)* | I: ≥1 prior line ET and ≤2 chemotherapy, prior CDK4/6i allowed  III: ≥1 prior line ET and ≤1 chemotherapy, no prior CDK4/6i in metastatic setting, no prior SERD, PI3K/AKT/mTOR inhibitor | Ib: Open label parallel group, dose-finding  III: Double-blind randomized, 1:1, 2-arm | Fulvestrant + Capivasertib + Palbociclib (expansion later to include other CKD4/6i)  Vs Fulvestrant + Placebo + Palbociclib | Ib: recommended phase 3 dose  III: PFS |
| VIKTORIA-1  NCT05501886 | III | 701* | 1-2 prior lines ET (including non-steroidal AI + CDK4/6i) and ≤1 chemotherapy; no prior PI3K/AKT/mTOR inhibitor | Parallel groups (*PIK3CA* WT and *PIK3CA* mut), each open-label randomized | Fulvestrant + Gedatolisib + Palbociclib  Vs Fulvestrant + Placebo + Gedatolisib  Vs Alpelisib + Fulvestrant (if *PIK3CA*mut) OR Fulvestrant alone (if *PIK3CA* WT) | PFS in patients with *PIK3CA* WT and *PIK3CA* mutant disease |
| Antibody Drug Conjugates | | | | | | |
| DESTINY-Breast06  NCT04494425 | III | 850* | 2 prior ET or PD in first 6 months of ET + CDK4/6i, no prior chemotherapy | Open-label randomized 1:1, 2-arm | Trastuzumab deruxtecan  Vs Physician’s choice chemotherapy (Capecitabine, Paclitaxel, Nab-paclitaxel) | PFS |
| DAISY  NCT04132960 | II | 186 (inclusive of all cohorts) | HER2 negative, HER2 low and HER2 positive cohorts. Patients with ER+ disease that is HER2 negative or HER2 low must have prior CDK4/6i and capecitabine | Single-arm 3-cohort study | Trastuzumab deruxtecan | Best Objective Response |
| TROPION-Breast01  NCT05104866 | III | 725 | Prior ET and 1-2 lines prior chemotherapy | Open-label randomized 1:1, 2-arm | Datopotamab deruxtecan  Vs Physician’s choice chemotherapy (Capecitabine, Gemcitabine, Eribulin, Vinorelbine) | PFS, OS |

Notes: *Anticipated total trial enrolment of trial still recruiting patients.

Abbreviations: AI = aromatase inhibitor, CDK4/6i = CDK4/6 inhibitor, ET = endocrine therapy, N = total patient enrolment, ORR = objective response rate; OS = overall survival, PFS = progression free survival, SERD = selective estrogen receptor degrader, SERM = selective estrogen receptor modulator
